# Molecular characterization of enterovirus detected in cerebrospinal fluid and wastewater samples in Monastir, Tunisia, 2014–2017

**DOI:** 10.1186/s12985-022-01770-w

**Published:** 2022-03-18

**Authors:** Yosra Rmadi, Aida Elargoubi, Rubén González-Sanz, Maha Mastouri, Maria Cabrerizo, Mahjoub Aouni

**Affiliations:** 1 Faculty of Pharmacy, Laboratory of Infectious Diseases and Biological Agents, University of Monastir, LR99-ES27, 5000 Monastir, Tunisia; 2 Laboratory of Microbiology, Fattouma Bourguiba University Hospital, Monastir, Tunisia; 3 Enterovirus and Viral Gastrointestinal Unit, National Centre for Microbiology, Instituto de Salud Carlos III, Madrid, Spain

**Keywords:** Enteroviruses, Meningitis, Wastewater, Phylogenetic analysis

## Abstract

**Background:**

Enteroviruses (EVs) are considered the main causative agents responsible for aseptic meningitis worldwide. This study was conducted in the Monastir region of Tunisia in order to know the prevalence of EV infections in children with meningitis symptoms. Detected EV types were compared to those identified in wastewater samples.

**Methods:**

Two hundred CSF samples collected from hospitalized patients suspected of having aseptic meningitis for an EV infection between May 2014 and May 2017 and 80 wastewater samples collected in the same time-period were analyzed. EV detection and genotyping were performed using PCR methods followed by sequencing. Phylogenetic analyses in the 3′-VP1 region were also carried-out.

**Results:**

EVs were detected in 12% (24/200) CSF and in 35% (28/80) wastewater samples. EV genotyping was reached in 50% (12/24) CSF-positive samples and in 64% (18/28) sewage. Most frequent types detected in CSF were CVB3, E-30 and E-9 (25% each). In wastewater samples, the same EVs were identified, but also other types non-detected in CSF samples, such as E-17,CVA9 and CVB1 from EV species B, and EV-A71 and CVA8 from EV-A, suggesting their likely lower pathogenicity. Phylogenetic analysis showed that within the same type, different strains circulate in Tunisia. For some of the EV types such as E-9, E-11 or CVB3, the same strains were detected in CSF and wastewater samples.

**Conclusions:**

Epidemiological studies are important for the surveillance of the EV infections and to better understand the emergence of certain types and variants.

## Background

Enteroviruses (EVs), which are members of the genus *Enterovirus* and belong to the *Picornaviridae* family, are small viruses with single positive-strand RNA genome with icosahedral capsid [1]. EV particles are constructed of 60 repeating protomersthat contain the viral genome which is a single RNA strand with two untranslated regions (5′ and 3′ -UTR) flanking a large open reading frame (ORF) which is processed to give rise to four structural proteins, VP1 to VP4, and non-structural proteins (2A to 2C and 3A to 3D). The VP1 capsid protein is the most external and immunodominant of the picornavirus capsid proteins and contains neutralization epitopes whereas the small protein VP4 is myristoylated and located on the inside of the virion. There are more than 100 EV types that infect humans and are spread mainly through fecal–oral route and via respiratory route with highest risk among children [2]. According to their molecular properties, the human EVs are classified into four distinct species: EV-A, B, C and D (Table 1) [3]. EVs are involved in many diseases of the central nervous system (SNC) such as aseptic meningitis, meningoencephalitis, encephalitis or paralysis, but they can also cause respiratory pathologies, neonatal sepsis-like disease, or hand, foot and mouth disease [4]. EVs are implicated in other diseases with heterogeneous presentations such as myocarditis, pleurodynia, pancreatitis, and hepatitis [5]. EVs are considered the main cause of aseptic meningitis, an illness characterized by serious inflammation of the linings of the brain that is not associated with any identifiable bacterial pathogen in the cerebrospinal fluid (CSF) [6].Some serotypes such as echovirus type 30, 6, 11 and 9 or coxsackievirus B5, are.more frequently associated with meningitis than others worldwide, especially in children [7–10]. EV infections can cause sporadic cases, outbreaks, and epidemics, such as those reported in China, Netherlands and Qatar in the last 10 years [11–13].

**Table 1:** Current classification of human EVs [3]

Species	Serotypes
A	Coxsackievirus A2–8, 10, 12, 14, 16 Enterovirus-A71, A76, A89-92,A114, A119-121
B	Coxsackievirus B1–6, A9 Echovirus 1–7, 9,11–21, 24–27, 29–33 Enterovirus B-69, B73–75, B77–88, B93, B97-98, B100-101, B106-107
C	Coxsackievirus A1, 11, 13, 17, 19, 20, 21, 22, 24 EnterovirusC95-96, C99, C102, C104-105, C109, C113, C116–118 Poliovirus 1–3
D	EV-D68 D70, D94, D111

In Tunisia, several studies about detection of EVs in neurological infections have been published with prevalence ranging from 9 to 33% [14, 15]. On the other hand, and due to their physical properties, EVs can persist in the environment for long periods especially in wastewater, and given that they are discharged in the sea, EV water contamination could be a serious problem for public health risk. Furthermore, EV detection in sewage has been frequently demonstrated [16–18] and is a useful tool for polio surveillance at the current stage of the global eradication especially in countries that have never stopped transmission of polio (Afghanistan and Pakistan) and those where the oral polio vaccine is used and vaccine-derived polioviruses can emerge causing outbreaks of paralytic polio [19, 20].

In order to better understand the epidemiology of EV infections in Monastir, Tunisia, in this collaborative study, prevalence of EVs in CSF samples from children with suspected viral neuro-meningeal infection and admitted to the Fattouma Bourguiba University Hospital in Monastir Tunisia between May 2014 and May 2017, was described. In addition, detected EVs were characterized and the sequences obtained were compared with those identified in wastewater samples collected in the same region during the same time-period.

## Methods

### Patients and clinical samples

Two hundred CSF samples from 200 children admitted to the University Hospital Fattouma Bourguiba of Monastir (Tunisia) between May 2014 and May 2017 with clinical suspicion of viral central nervous system (CNS) infection were included. Bacteria and fungi infections were ruled out (tested by routine hospital procedures). Epidemiological and clinical symptoms data were retrospectively collected from medical records. CSF samples were stored at − 80 °C until processing. The study and the data collection procedure were approved by the Ethics and Research Committee of the Fattouma-Bourguiba Public Hospital.

### Wastewater samples

Eighty samples of raw influent and treated wastewater were collected between 2014 and 2017 from two sewage treatment plants: Sayada-Lamta-Bouhjar (STP1) and Elfrina (STP2). STP1, located in the area of Monastir, was created in 1993 and receives domestic wastewater from three cities, Sayada, Lamta and Bouhjar. Its average daily volume is 2160 m^3^ per day. STP2, constructed in 1995, is located in a coastal region and receives two kinds of water, domestic and industrial water; the daily flow average is 13500 m^3^ per day. In both plants, activated sludge is used for the treatment process. Samples (1L) were collected twice per month, transferred to the laboratory into a cool box, and were stored at + 4 °C until processing. In water samples, viruses are mostly present in low or very low concentrations, thus, it is necessary to concentrate the samples before analyses. In our study we used the adsorption/elution method based on precipitation with beef extract and polyethylene glycol (PEG) 6000, as described previously [21].

### EV detection and type characterization

EV detection and genotyping were performed in the Spanish Enterovirus Reference Laboratory (National Centre for Microbiology, Instituto de Salud Carlos III, Madrid, Spain).

Viral RNA was extracted from 140 µl of CSF and wastewater samples using QIAamp viral RNA kit (Qiagen) according to manufacturer’s instructions. The purified RNA was stored at 80 °C until amplification assay. EV detection was performed by conventional RT-nested PCR in the high conserved 5’non-coding region (310 bp fragment), as described by Casas et al. [22].

EV-positive samples were genotyped by amplification of partial 3′-VP1 region and sequencing. According to the literature, EV types from species A and B are the main causing aseptic meningitis. Then, two RT-PCRs specific for EV-A and EV-B previously published [23] were used in CSF samples while in wastewater samples, RT-PCRs for EV-A, B, C [23] together with another RT-PCR for EV-D68 [24] were performed. PCR products were purified using illustraExoProStar^TM^1-Step and sequenced using a BigDye Terminator kit (Applied Biosystems, Foster City, CA, USA) and the inner nested PCR primers. Partial VP1 (400 bp) sequences obtained were compared with other EV sequences available in GenBank using basic local alignment BLAST (http://www.ncbi.nlm.nih.gov/BLAST) and were assigned to the serotype of the strain that gave the highest identity score (> 75%).

For phylogenetic analyses, multiple 3′-VP1 sequence alignments were performed by the Clustal W program. Trees were constructed using the Neighbour-Joining method, with the Maximum Likelihood distance model and the Boot-trap method (1000 pseudo-repeats), implemented in MEGA software version 7.0 [25].

### Statistical analysis

All statistical tests were analyzed using SPSS statistics software version 28.0. Data were presented as Mean ± Standard Deviation (SD) for continuous variables and a P-value less than 0.05 was considered statistical differences (P < 0.05).

## Results

### EV detection and typing in CSF samples

**Fig. 1:**
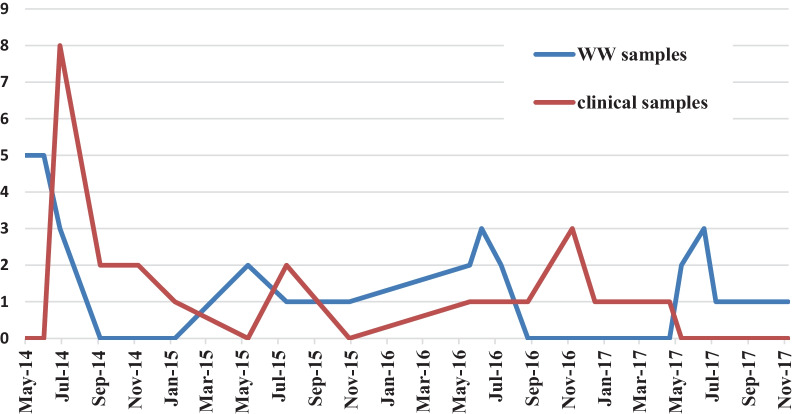
Monthly distribution of EV infections detected in clinical and wastewater (ww) samples from this study

EV was positive in 24 out of 200 CSF samples tested, supposing a prevalence of 12% during the 3 years-study period. Half of the EV infections were detected between May and August (12/24) and 29% (7/24) from September to December (Fig. 1).

Mean age of the EV-positive patients was 2.3 ± 2.8 years, ranging between 1 month and 12 years, being 58% boys and 41% girls (14/10). Fever, vomiting and headache were the most common clinical manifestations presented by the infected children (Table 2).

**Table 2:** Epidemiological and virological characteristics of the EV-infected patients included in this study

Laboratory code	collection date	Age*	Sex	main symptoms	EV type
CSF/03_07_2014	03/07/2014	1 mo	M	Fever/vomiting	E-30
CSF/19_05_2016	19/05/2016	2 yr	M	Fever/headache/vomiting	CVB3
CSF/14_07_2014	14/07/2014	2 mo	M	FEVER/ headache	E-9
CSF/12_01_2015	12/01/2015	2 mo	F	Fever/headache/vomiting	E-9
CSF/02_07_2014	02/07/2014	1 yr	F	Fever/stiff neck	E-9
CSF/08_09_2014	08/09/2014	2 yr	M	Fever/headache	Non-typed
CSF/17_11_2016	17/11/2016	1 mo	F	Fever/vomiting	Non-typed
CSF/15_07_2014	15/07/2014	2 yr	M	Fever/vomiting	E-30
CSF/22_11_2016	22/11/2016	1.5 yr	M	Fever/headache	CVB3
CSF/12_11_2016	12/11/2016	2 yr	M	Fever/vomiting/headache	E-30
CSF/25_04_2017	25/04/2017	3 yr	F	Fever/vomiting	CVB5
CSF/01_07_2016	01/07/2016	2 yr	M	Fever/headache/vomiting	E-11
CSF/25_07_2015	25/07/2015	3 yr	M	Fever/headache/vomiting	CVB3
CSF/30_06_2016	30/06/2016	4 yr	M	Fever/headache	E-11
CSF/04_07_2014	04/07/2014	7 yr	F	Fever/headache/stiff neck	Non-typed
CSF/11_11_2014	11/11/2014	1 mo	M	Fever/headache/vomiting	Non-typed
CSF/08_09_2014	08/09/2014	5 mo	M	Fever/headache	Non-typed
CSF/20_12_2016	20/12/2016	5 mo	F	Fever/vomiting/headache/stiff neck	Non-typed
CSF/09_07_2014	09/07/2014	12 yr	M	Fever/vomiting	Non-typed
CSF/10_07_2014	10/07/2014	3 mo	F	Fever/headache	Non-typed
CSF/08_07_2014	08/07/2014	3 yr	M	Fever/headache/vomiting	Non-typed
CSF/05_11_2014	05/11/2014	2.5 yr	F	Fever/vomiting	Non-typed
CSF/13_07_2015	13/07/2015	3 mo	F	Fever/vomiting/headache/stiff neck	Non-typed
CSF/29_08_2016	29/08/2016	7 yr	F	Fever/vomiting/	Non-typed

*mo: months; yr: years

EV genotyping was reached in 50% (12/24) of the positive samples. Five different EV types were identified, all belonging to EV-B species. Echovirus (E-30), coxsackievirus (CV) B3 and E-9 were the most frequent EV detected (three samples each, 25%), followed by E-11 (N = 2, 17%) and CVB5 (N = 1, 8%). Non-typed EVs were confirmed by sequencing the 5`-NCR PCR products, being all of them from species B.

### EV detection and typing in wastewater samples

80 wastewater samples were analyzed, 40 from STP1 and 40 from SPT2. EV genomes were detected in 35% (28/80) samples, 42% (17/40) of the raw sewage samples and in 27% (11/40) of treated ones (Table 3). Figure 1 showed the seasonal distribution of detected EV.

**Table 3:** Characteristics of the wastewater EV-positive samples and genotyping results

Wastewater sample	Collection date	STP	Origin	EV type
WW1/05_05_2014	05/05/2014	1	R	EV-A71
WW1/05_07_2016	05/07/2016	1	R	CVB3
WW2/15_07_2016	15/07/2016	2	R	CVB3
WW2/23_05_2016	23/05/2016	2	T	CVB3
WW2/12_07_2017	12/07/2017	2	R	Non-typed
WW2/11_11_2017	11/11/2017	2	T	Non-typed
WW2/05_06_2014	05/06/2014	2	T	Non-typed
WW1/22_05_2014	22/05/2014	1	R	Non-typed
WW1/22_05_2017	22/05/2017	1	T	CVB1
WW1/12_06_2017	12/06/2017	1	R	EV-A71
WW1/12_06_2016	12/06/2016	1	T	Non-typed
WW1/19_06_2014	19/06/2014	1	R	E-9
WW1/22_06_2016	22/06/2016	1	R	E-11
WW1/15_05_2017	15/05/2017	1	R	Non-typed
WW1/15_05_2015	15/05/2015	1	T	E-11
WW1/27_05_2014	27/05/2014	1	R	E-17/CVA9
WW2/22_06_2014	22/06/2014	2	R	E-11/E-30
WW2/06_06_2016	26/06/2016	2	T	E-11
WW1/19_06_2017	19/06/2017	1	R	Non-typed
WW1/27_05_2015	27/05/2015	1	R	CVA9
WW1/19_07_2015	19/07/2015	1	T	CVB3
WW1/02_11_2015	02/11/2015	1	T	Non-typed
WW1/12_06_2014	12/06/2014	1	T	E-17
WW1/08_05_2014	08/05/2014	1	R	CVA8
WW1/05_07_2014	05/07/2014	1	T	Non-typed
WW1/22_06_2017	22/06/2017	1	R	CVB1
WW1/15_05_2016	15/05/2016	1	R	Non-typed
WW1/02_07_2014	02/07/2014	1	R	E-17/CVA9

R: Raw wastewater sample; T: treated wastewater sample; 1: STP1; 2:STP2

EV were typed in 64% (18/28) positive-sewage samples. Nine different EV types were detected, CVB3 and E-11 (22% each), E-17 and CVA9 (16% each), CVB1 (11%), E-9 (5%) and E-30 (5%) from EV species B, and EV-A71 (11%) and CVA8 (5%) from EV-A.A mixed contamination by two different EV was found in three samples.

### Phylogenetic analyses and correlation between the human and environmental detected EV strains

**Fig. 2:**
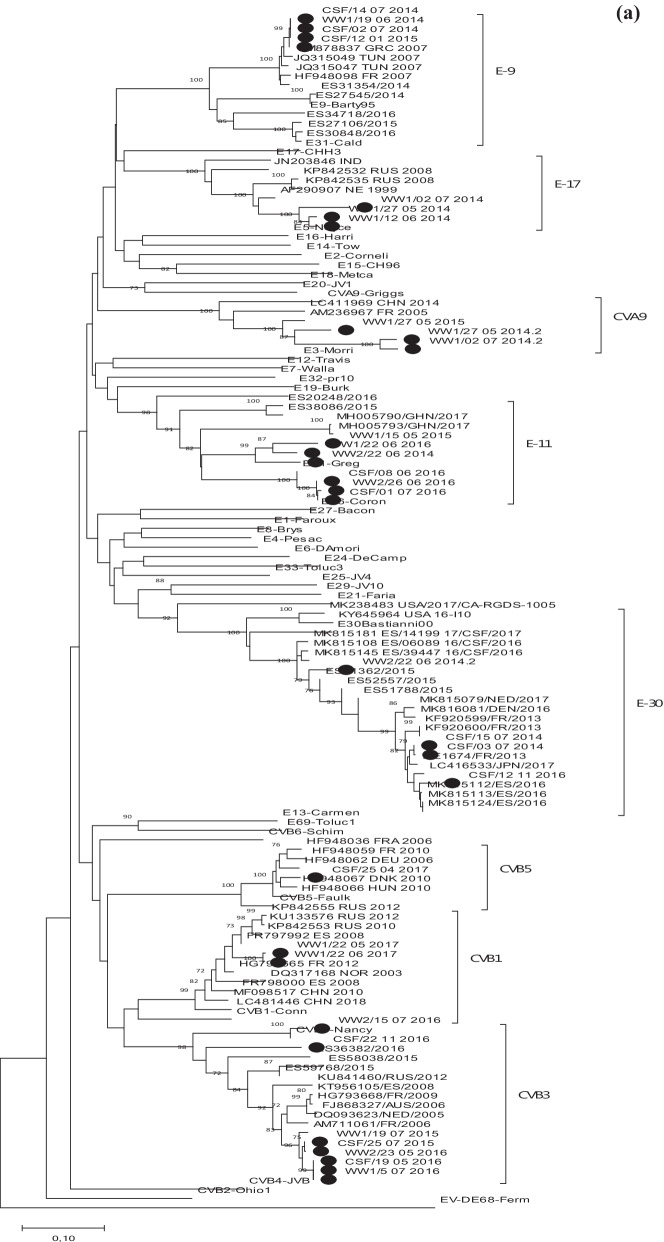

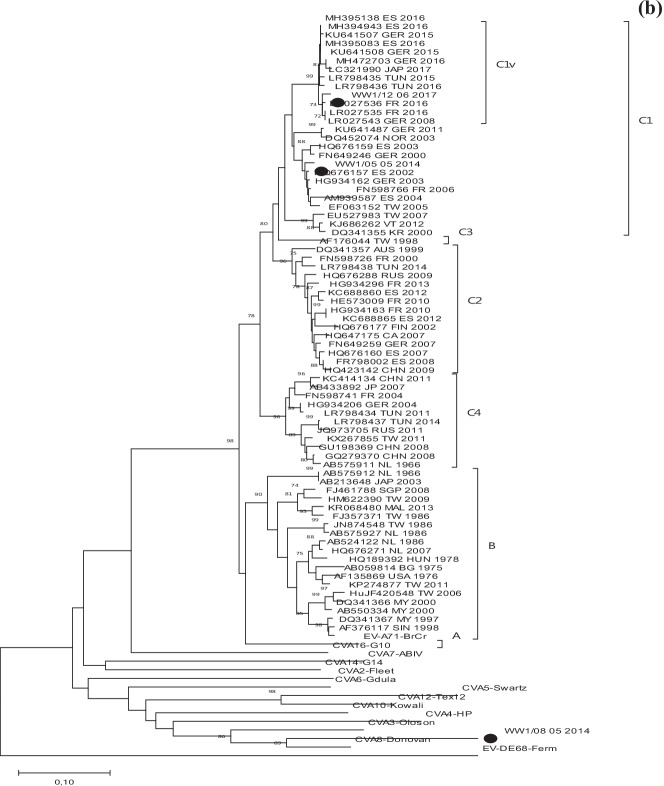
Phylogenetic trees of EV-B (**a**) and EV-A (**b**) sequences isolated in this study, prototype strains and others available in GenBank based on 3′-VP1 gene sequence. Tunisian strains isolated in this study are indicated with black circle. The trees were rooted with the EV-D68 Fermon sequence. Only bootstraps > 70 are indicated

In order to confirm the results obtained with BLAST analysis and to identify the genetic relationships between clinical and environmental sequences detected in this study and with other EV strains available in GenBank in the same 3’VP1 region, two phylogenetic analyses were performed, one for EV-A and other for EV-B sequences. All sequences obtained in this study were grouped with their respective prototype strain and other sequences that belonged to the assigned EV type (Fig. 2a, b). EV-B phylogenetic analysis showed that all E-30 identified in CSF samples and sewage samples from 2014 and 2016 clustered with other strains isolated worldwide between 2013 and 2017. However, strains CSF/15_07_2014 and CSF/03_07_2014 had higher similarity with CSF/12_11_2016 than with the E-30 strain isolated in wastewater during the same year, WW2/22_06_2014 (96 vs. 94%) (Fig. 2a).CVB3 was the most common serotype identified in this study (3 in CSF and 4 in wastewater samples). Phylogenetic tree showed that the five Tunisian strains from 2015 and 2016 grouped together with other European and non-European strains isolated during 2005 and 2012. However, they were separated from WW2/15_07_2016 strain, which was closely related to prototype strain Nancy (99%), and from CSF/22_11_2016 sequence, which revealed the highest divergence (13–28%). The strains circulating during July 2015 and May–July 2016 were detected both in environmental and clinical samples.

All Tunisian E-9 strains detected in 2014 and 2015 (N = 4), in CSF as well as in wastewater samples, belonged to the same cluster and were more related to French, Greek and Tunisian sequences detected in 2007 than to those circulating in Spain between 2014 and 2016.

Regarding E-11 strains, they fell into two clusters, one formed by CSF and wastewater sequences from 2016 and the other by those identified in environmental samples in 2014, 2015 and 2016. All shared a homology of 71–75% with strains identified in stool samples from Ghana during 2017.

Finally, for E-17, CVB1 and CVA9, with 2–3 sequences only identified in wastewater samples, the tree showed an only circulating strain in the period of study (Fig. 2a).

Figure 2b showed EV-A phylogenetic analysis performed with 12 prototype strains, those EV-A71 and CVA8 sequences from this study (detected only in sewage) and several EV-A71 from other countries available in GenBank. Resulting tree revealed that both Tunisian EV-A71 strains belonged to the subgenogroup C1. However, while WW1/5_055_2014 sequence was closely related to older strains isolated between 2000 and 2008, WW1/12_06_2017 belonged to the cluster formed by sequences of the EV-A71 C1 variant first identified in Germany in 2015. This was the causative agent of a large encephalitis outbreak that occurred in Spain during 2016. Other Tunisian strains from 2015 and 2016 reported in previous studies, also grouped in this cluster.

## Discussion

The present study describes the prevalence of EVs associated with aseptic meningitis in children from the Monastir region between 2014 and 2017. EV infection was confirmed in 12% of the studied cases by-PCR techniques. Previous studies in other countries reported higher prevalence, ranging from 44 to 76% [10, 13, 26]. However, prevalence similar to or lower than ours have also been reported in Palestine (18%), Germany (4%) and Italy (4%) [27–29].Regarding previous studies performed in Tunisia, they showed higher prevalence than ours. A study conducted in 2002 by Gharbi et al., EVs were responsible for 71% neurological infections in children from the Monastir region [30]. In 2007, EV was isolated in 33% of CSF samples from children [14]. Only one recent report demonstrated similar prevalence since EVs were responsible for only 10% of aseptic meningitis cases in children (14/143) admitted into two Tunisian hospitals between 2011 and 2013 [15].Overall, variation in the epidemiological results from study to another can be explained by differences in methodology, that is, type of PCR assay (real-time RT-PCR, conventional RT-PCR, multiplex RT-PCR), primers used or genomic region amplified. The age of the patients, the time of sampling from the onset of symptoms and the temperature and time of sample storage can also influence the sensitivity in the detection of EV infections. Specifically, the low occurrence of EV detection in our series could be attributed to a low viral load in our samples due to the preservation and transport process but also to the epidemic circulation pattern characteristic of the different types of EV, with years of high incidence followed by others with no or very low detection. Furthermore, other pathogens causing aseptic meningitis such as Herpes virus simplex 1 and 2, West Nile Virus and Toscana virus could be implicated [31].

In addition, Kupila et al. showed that detection of EV by PCR in CSF samples was high during the early course of enteroviral meningitis disease, which means that inadequate sample collection can influence the results [32]. Unfortunately, in this study, the number of days between symptom onset and sampling was not available. In line with previous studies [9, 12], our findings showed that the incidence of aseptic meningitis by EV in males is higher than in females, although the difference is not statistically significant. As shown in previous studies, most cases of aseptic meningitis were observed in summer and autumn. The peak seasonality of EV is consistent with those reported in countries with temperate climates [10, 15, 27, 33]. The percentage of detected EV successfully genotyped varies from one work to another according to the technique and the sample type used. In the present study, EV genotyping was successful in 50% of the positive CSF samples. It is lower than those published by other authors [28, 34], but slightly lower than that reported by the Spanish laboratory itself [8, 23]. This could be explained by degradation of RNA during the transport of the samples with probable cycles of freezing/unfreezing or mispairing of the primers used.

Globally, EV from species B were the most frequent types detected in aseptic meningitis, although others such as CV-A6 or EV-A71 can be implicated [35]. In our series, only EV-B were detected in CSF samples. E-30, E-9 and CVB3 were the most prevalent, but E-11 and CVB5 were also detected. Meningitis associated with E-30 and E-9 was reported worldwide, as well as E-11 [8, 14, 36, 37].

In Tunisia, a study conducted by Bahri et al., during a 12 –year period, demonstrated that E-30, E-11 and E-6 were the most frequently EV isolated every year [33]. CVB3 was also reported in meningitis cases, sometimes associated with epidemic outbreaks [38, 39]. Finally, CVB5 was also involved in cases of aseptic meningitis in countries such as France and China [12, 40]. Environmental EV detection has complemented poliovirus surveillance within the Global Polio Eradication Initiative (GPEI) for years [19, 20], but it is also a useful tool for measuring viral contamination in water. EVs have been isolated from many types of water especially wastewater, river or seawater, drinking, and swimming pool water [16, 41–43].

In this study, the presence of EVs in wastewater was monitored during the same period in order to correlate the strains found in the environment with those causing aseptic meningitis in Monastir. Detection rate of EVs in wastewater (35%) was similar to other results described in China, Romania and Greece [44–46]. In addition, our study showed the presence of EVs not only in raw but also in treated wastewater (in almost 30% of these samples). Other studies also documented higher detection rate of EVs from both raw and treated wastewater [47, 48],

indicating that the purification treatments are not always effective against these viruses that can survive in a long period of time in wastewater due to their highly resistant properties and thermal stability [49].

EV prevalence in wastewater samples was higher than in CSFs. Furthermore, EV detection in sewage appears to precede that in clinical samples, as shown in Fig. 1. Although the most frequent EVs detected in sewage were from species B, EV-A were also found. Several types such as CVB3, E-9, E-11 and E-30, were detected both in clinical and wastewater samples, indicating a correlation between clinical cases and the excretion by the population, but other EV-B (CVB1, CVA9 and E-17) and two EV-A (EV-A71 and CVA8) were only identified in wastewater samples. These discrepancies in detection between clinical and water samples might be due to a silent circulation of EV but also to the fact that in the present study, only CSF samples from meningitis cases were included. With respect to the first hypothesis, most of the studies of environmental surveillance revealed the presence of those EV types that are causing human infections [17, 18], but also other types that are circulating asymptomatically [50, 51].

Because EV-A71 and CVA8, just like any EV, can cause aseptic meningitis but frequently, both types are associated with muco-cutaneous pathologies such as HFMD or non-specific exanthemas [35, 52, 53]. EV-A71, in addition, have been responsible for large outbreaks worldwide with subsequent severe neurological complications [54]. Then, EV-A71 and CVA8 detection only in sewage can be related to asymptomatic circulation of the infections and/or with the fact that different types of clinical samples associated with diseases other than meningitis were not studied. It has been demonstrated that for the diagnosis of some pathologies such as encephalitis or HFMD, the appropriate sample is not CSF but respiratory or stool samples [55]. This is the first CVA8 detection in sewage from Tunisia; it is not one of the most frequently detected EV types, although it has been reported in other environmental surveillance studies [43, 56]. Regarding EV-A71, the two strains isolated in this study belonged to the subgenogroup C1. EV-A71 subgenogroup C1 and C2 have been frequently reported in Europe associated with neurological symptoms [57, 58]. Furthermore, one of the detected strain in Monastir in 2017 was closely phylogenetically related to the EV-A71 C1variant detected firstly in Germany in 2015 [59] and then in other European countries, including Spain, where in 2016 it caused an important encephalitis outbreak [60]. Surprisingly, the phylogenetic analysis revealed that this variant have already been circulating in Tunisia since 2015.

Finally, a phylogenetic tree constructed with detected EV-B showed that within the same type, different strains circulate in Tunisia. For E-9, E-11 or CVB3 the same strain circulates in different years; for E-30 different strains co-circulate at the same time. Furthermore, in some of them, the same strains causing meningitis are detected in water. Unfortunately, not too many EV sequences from African countries in the same 3’-VP1 region are available in the GenBank to determine whether the strains circulating in Tunisia are more closely related to European strains than to those from Africa.

With regard to the limitations of our study, the low number of samples and preservation problems of them during transport to Madrid may have led to a bias in the results.

## Conclusions

In order to perform a good surveillance of EV infections in Tunisia, it is necessary to increase the number of clinical samples studied as well as the types of specimens appropriate to the different clinical syndromes caused by these viruses. Moreover, establishing an environmental surveillance system would also provide a lot of information on which EVs are circulating asymptomatically, which could lead to outbreaks of public health relevance. Both types of surveillance are complementary and allow us to improve our knowledge about the epidemiology and the molecular evolution of these viruses.

## Data Availability

All data generated or analyzed during this study are included in this article.

## Abbreviations

E30: Echovirus 30
CNS: Central Nervous System
CSF: Cerebrospinal fluid
CVA8: Coxsackie virus A8
CVA9: Coxsackie virus A9
CVB1: Coxsackie virus B1
CVB3: Coxsackie virus B3
CVB5: Coxsackie virus B5
E11: Echovirus 11
E17: Echovirus 17
E9: Echovirus 9
EV: Enterovirus
EV-A: Enterovirus A
EV-A71: Enterovirus A71
EV-B: Enterovirus B
EV-D68: Enterovirus D68
F: Female
GPEI: Global Polio Eradication Initiative
HFMD: Hand, foot, and mouth disease
M: Male
NCR: Non-coding region
ORF: Open Reading Frame
PEG: Polyethylene glycol
R: Raw
RT-PCR: Reverse transcription polymerase chain reaction
STP: Sewage Treatment Plants
T: Treated
UTR: Untranslated region
VP1: Viral capsid protein 1
VP2: Viral capsid protein 2
VP3: Viral capsid protein 3
VP4: Viral capsid protein 4
